# Supplementary material for: Comprehensive structure-function characterization of DNMT3B and DNMT3A reveals distinctive de novo DNA methylation mechanisms
Source: Nat Commun. 2020 Jul 3;11:3355. doi: 10.1038/s41467-020-17109-4 (PMC7335073; doi:10.1038/s41467-020-17109-4)
Supplement: Supplementary file 1 — Supplementary Information [file 41467_2020_17109_MOESM1_ESM.pdf]

Supplementary Information

Comprehensive structure-function characterization of DNMT3B and DNMT3A reveals distinctive *de novo* DNA methylation mechanisms

Linfeng Gao, Max Emperle, Yiran Guo, Sara A Grimm, Wendan Ren, Sabrina Adam, Hidetaka Uryu, Zhi-Min Zhang, Dongliang Chen, Jiekai Yin, Michael Dukatz, Hiwot Anteneh, Renata Z. Jurkowska, Jiuwei Lu, Yinsheng Wang, Pavel Bashtrykov, Paul A Wade, Gang Greg Wang, Albert Jeltsch, Jikui Song

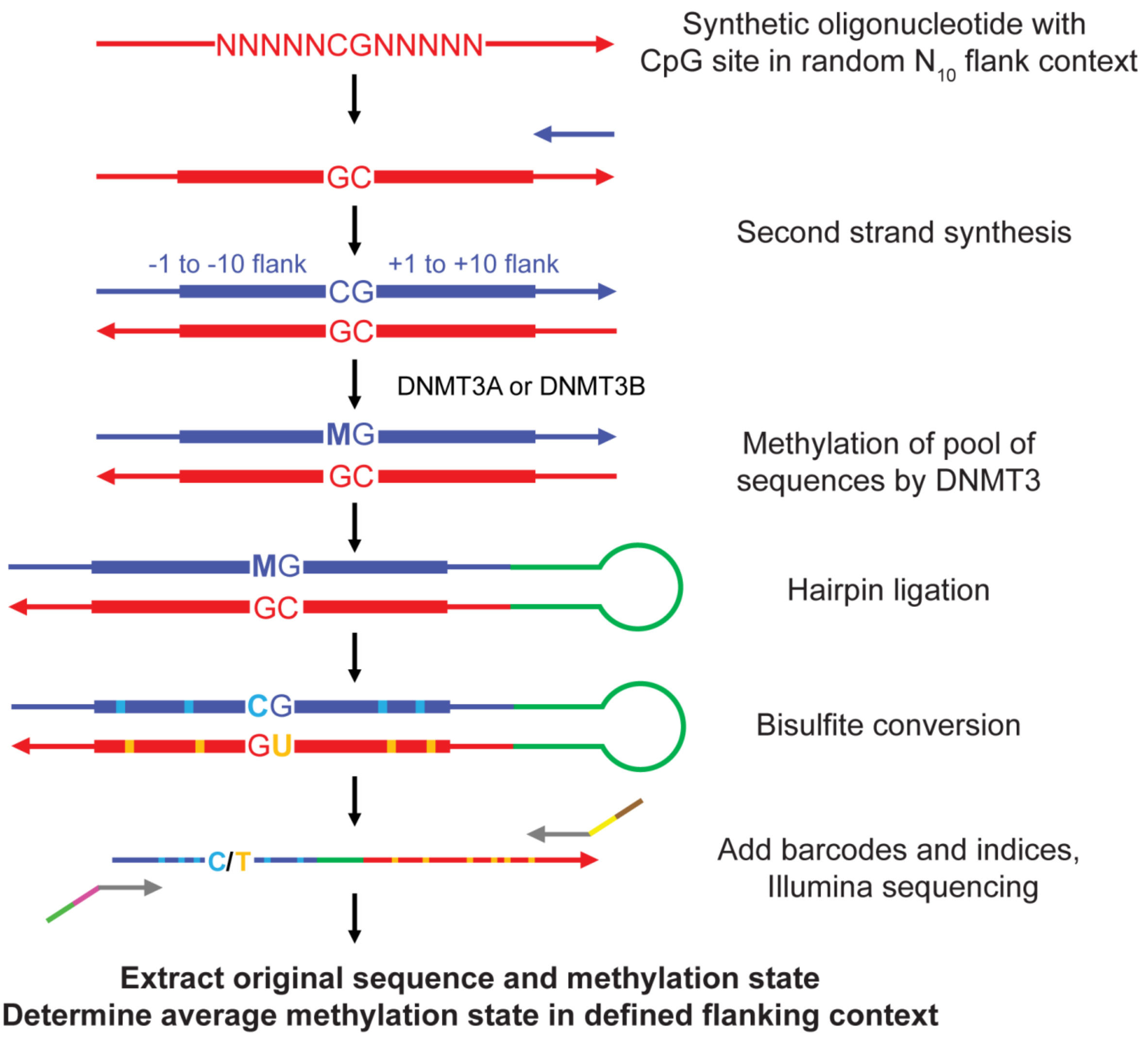

Supplementary Figure 1. Deep enzymology workflow allowing the methylation of pools of DNA substrates containing target sites in randomized sequence contexts. Pools of DNA substrates with a target CpG site in a randomized flanking context were generated using a synthetic oligonucleotide, which contains one target CpG site flanked by 10 random nucleotides on each side as starting point. In addition, randomized oligonucleotides were used containing the target cytosine followed by 5-methylcytosine (allowing to study the methylation preferences in the context of hemimethylated CpG sites) and in CpH (H=adenine, thymine, or cytosine) or CpN (N=adenine, guanine, thymine, or cytosine) context. Libraries of double stranded DNA substrate molecules containing CpG (or variants thereof) sites in randomized flanking context were then prepared by primer extension. The purified double stranded substrate library was incubated with DNMT3A or DNMT3B in methylation buffer containing AdoMet leading to the methylation of the target sites, depending on the enzymes' preferences for the respective flanking sequence. After stopping the methylation

reactions, a hairpin was ligated to the DNA substrates. Next, bisulfite conversion was carried out which converts cytosine to uracil, but leaves 5-methylcytosine intact. This was followed by amplification with primers specific for the converted DNA. Libraries for Illumina Next Generation Sequencing (NGS) were generated in a two-step PCR approach adding barcodes and indices. After determination of concentrations and validation of the products on acrylamide gels, the products from different methylation reactions were pooled in appropriate ratios and analyzed by Illumina NGS. Data were generated in independent repeats and sequenced at great depth (Supplementary Table 1). Control experiments without enzyme were conducted to determine the efficiency of the bisulfite conversion (Supplementary Table 1). Afterwards, the reads were filtered for duplicates and sequencing errors. For each read, the original flank sequence and the methylation state was extracted and compiled in a database. Later average methylation levels in different flanking sequence contexts were extracted, for example in CN, CNN, NNCGNN or NNNCGNNN contexts.

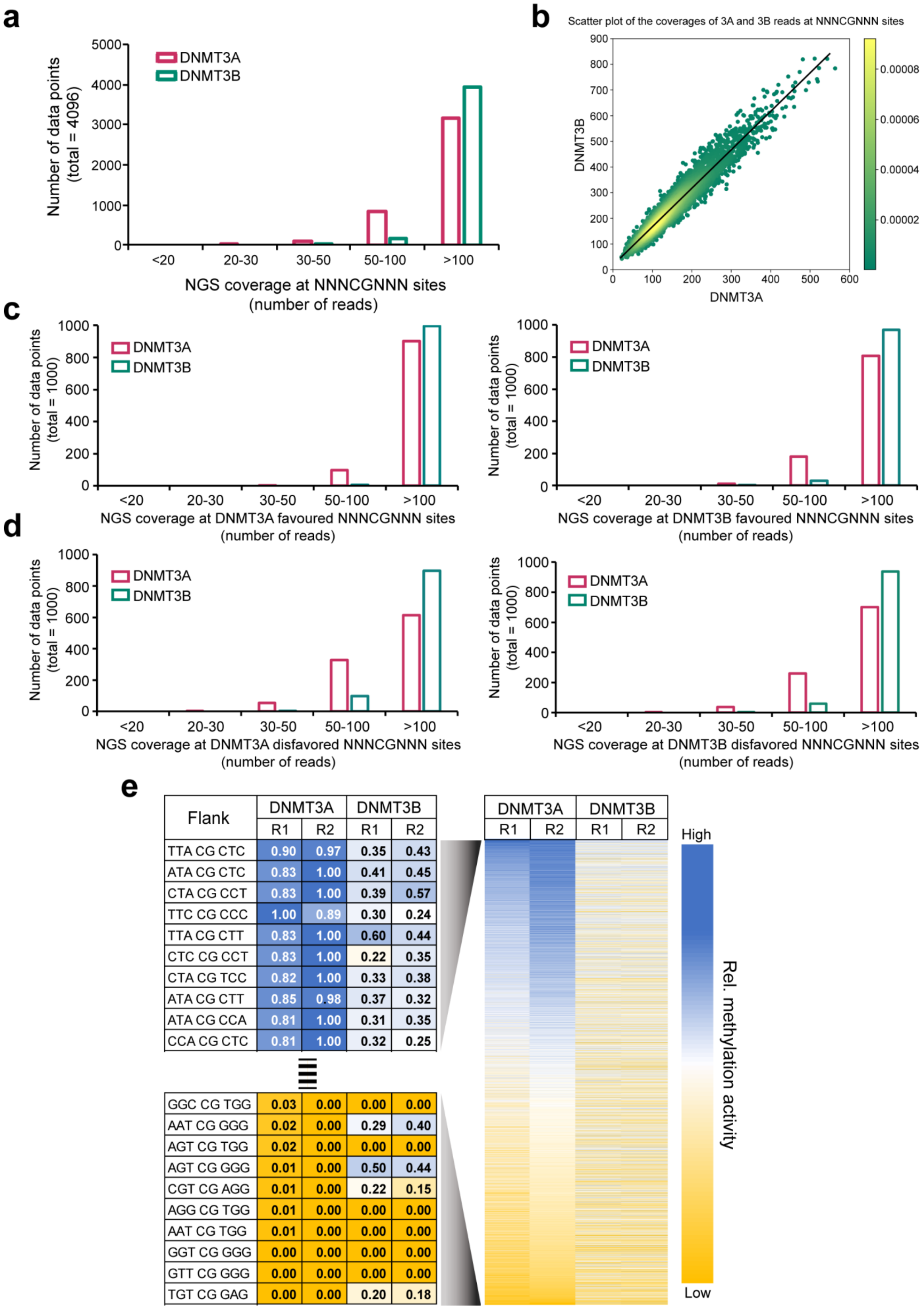

Flank	DNMT3A		DNMT3B	
	R1	R2	R1	R2
TTA CG CTC	0.90	0.97	0.35	0.43
ATA CG CTC	0.83	1.00	0.41	0.45
CTA CG CCT	0.83	1.00	0.39	0.57
TTC CG CCC	1.00	0.89	0.30	0.24
TTA CG CTT	0.83	1.00	0.60	0.44
CTC CG CCT	0.83	1.00	0.22	0.35
CTA CG TCC	0.82	1.00	0.33	0.38
ATA CG CTT	0.85	0.98	0.37	0.32
ATA CG CCA	0.81	1.00	0.31	0.35
CCA CG CTC	0.81	1.00	0.32	0.25
GGC CG TGG	0.03	0.00	0.00	0.00
AAT CG GGG	0.02	0.00	0.29	0.40
AGT CG TGG	0.02	0.00	0.00	0.00
AGT CG GGG	0.01	0.00	0.50	0.44
CGT CG AGG	0.01	0.00	0.22	0.15
AGG CG TGG	0.01	0.00	0.00	0.00
AAT CG TGG	0.01	0.00	0.00	0.00
GGT CG GGG	0.00	0.00	0.00	0.00
GTT CG GGG	0.00	0.00	0.00	0.00
TGT CG GAG	0.00	0.00	0.20	0.18

Supplementary Figure 2. Additional data regarding deep enzymology flanking sequence preferences. (a,b) Bar (a) and Scatter (b) plots showing the coverage of all 4096 NNNCGNNN sequences for NGS analysis of combined data from two consistent replicates. **(c)** Bar plots showing the coverage of the top 1000 NNNCGNNN sites most favored by mDNMT3A (left) or mDNMT3B (right). **(d)** Bar plots showing the coverage of the top 1000 NNNCGNNN sites most disfavored by mDNMT3A (left) or mDNMT3B (right) based on combined data from two consistent replicates. **(e)** Heatmap of the activities of mDNMT3A and mDNMT3B in 4096 different NNNCGNNN flanking contexts in two experiments showing the close correlation of the experimental repeats of both data sets and the pronounced differences between mDNMT3A and mDNMT3B. Data are sorted for DNMT3A activity. The left part shows an enlargement of the first and last 20 lines of the heatmap also including the corresponding sequences and normalized activity levels.

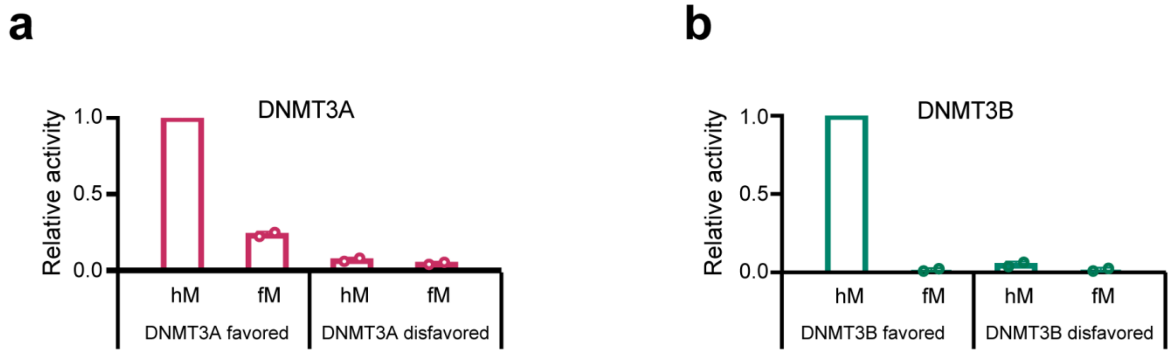

Supplementary Figure 3. Validation of the deep enzymology experiments. (a-b) Enzymatic validation of DNMT3A (a) or DNMT3B (b) on favored and disfavored substrates, using the MTase domains of mDNMT3A and mDNMT3B analyzed by radioactive methylation assays. For both enzymes, the 4096 NNNCGNNN flanks were sorted by preferences as indicated in Supplementary Fig. 2e. For mDNMT3A, the TTACGCCC (rank 49) and AGTCGTGG (rank 4014) sites were selected as favored and disfavored substrates, respectively, and subjected to methylation assays. For mDNMT3B, the CTACGGCT (rank 330) and AGTCGCAA (rank 3328) sites were selected as favored and disfavored substrates. Each substrate was used in hemimethylated form and with fully methylated CpG site to control for methylation of cytosine residues at other places of the substrate. The specific methylation of the CpG in the upper DNA strand is given by the difference of the activities observed on the hemi- and fully methylated substrates. Average values based on two experimental repetitions are displayed.

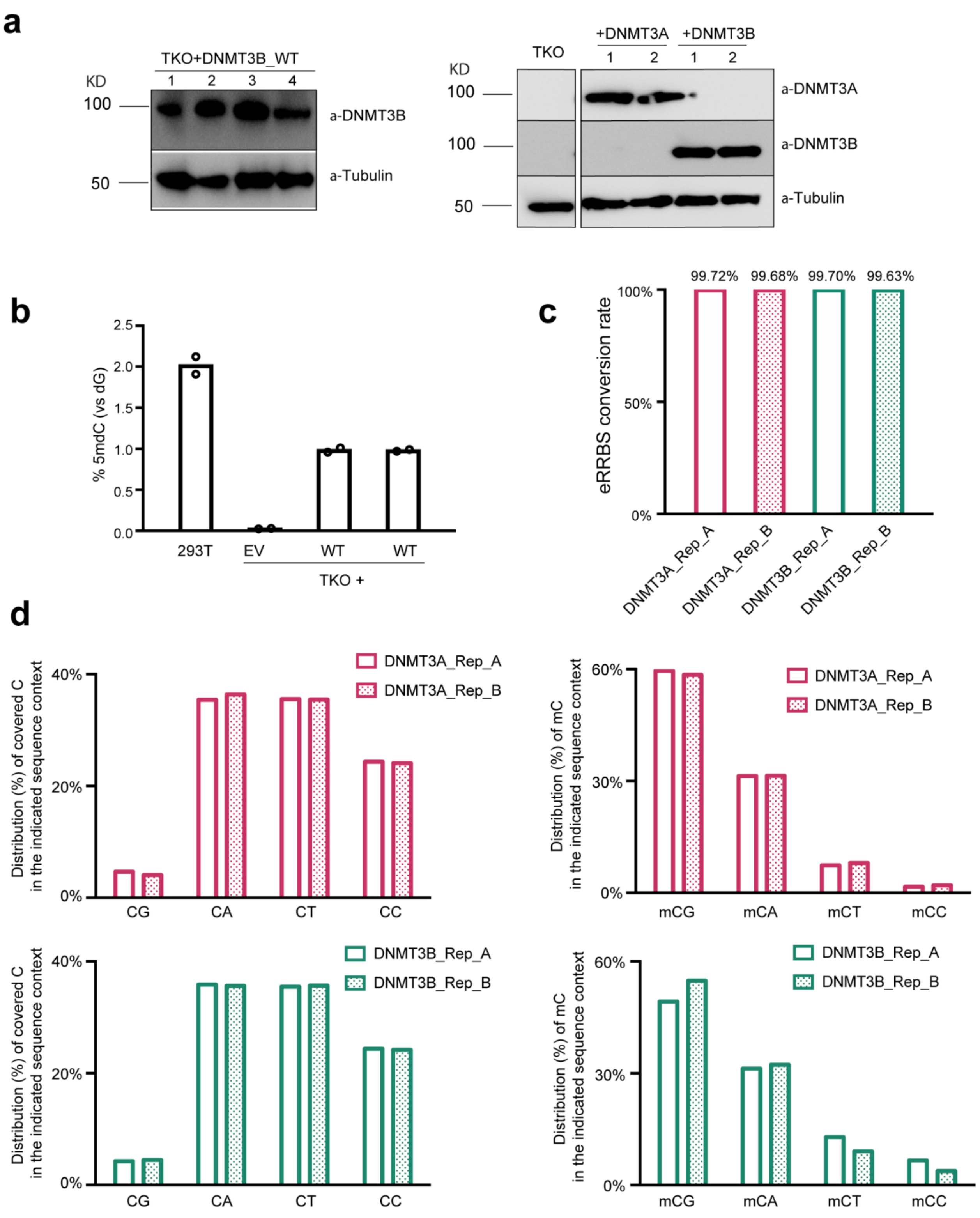

Supplementary Figure 4. Additional data regarding eRRBS-based methylation profiles of DNMT3B and DNMT3A. (a) Immunoblotting of hDNMT3B and hDNMT3A post-transduction into TKO ES cells reveals their similar expression levels in multiple

stable cell lines used for follow-up studies. TKO cells serve as negative. EV, empty vector. **(b)** LC–MS analysis reveals the global 5-methyl-2′-deoxycytidine (5-mdC) levels (indicated as 5-mdC/2′-deoxyguanosine ratio on the y-axis) in the TKO ES cells after stable transduction of empty vector or hDNMT3B (n = 2 replicates). **(c)** Bisulfite conversion rates observed in the independent eRRBS samples as determined by the unmethylated lambda DNA spike-in control. **(d)** eRRBS-based methylome profiling shows distribution of the total number of mapped C sites (two left panels) and methylated C (two right panels) with the indicated CpN sequence context in each biological replicate (rep A and B) of TKO lines rescued with either DNMT3A (two upper panels) or DNMT3B (two bottom panels).

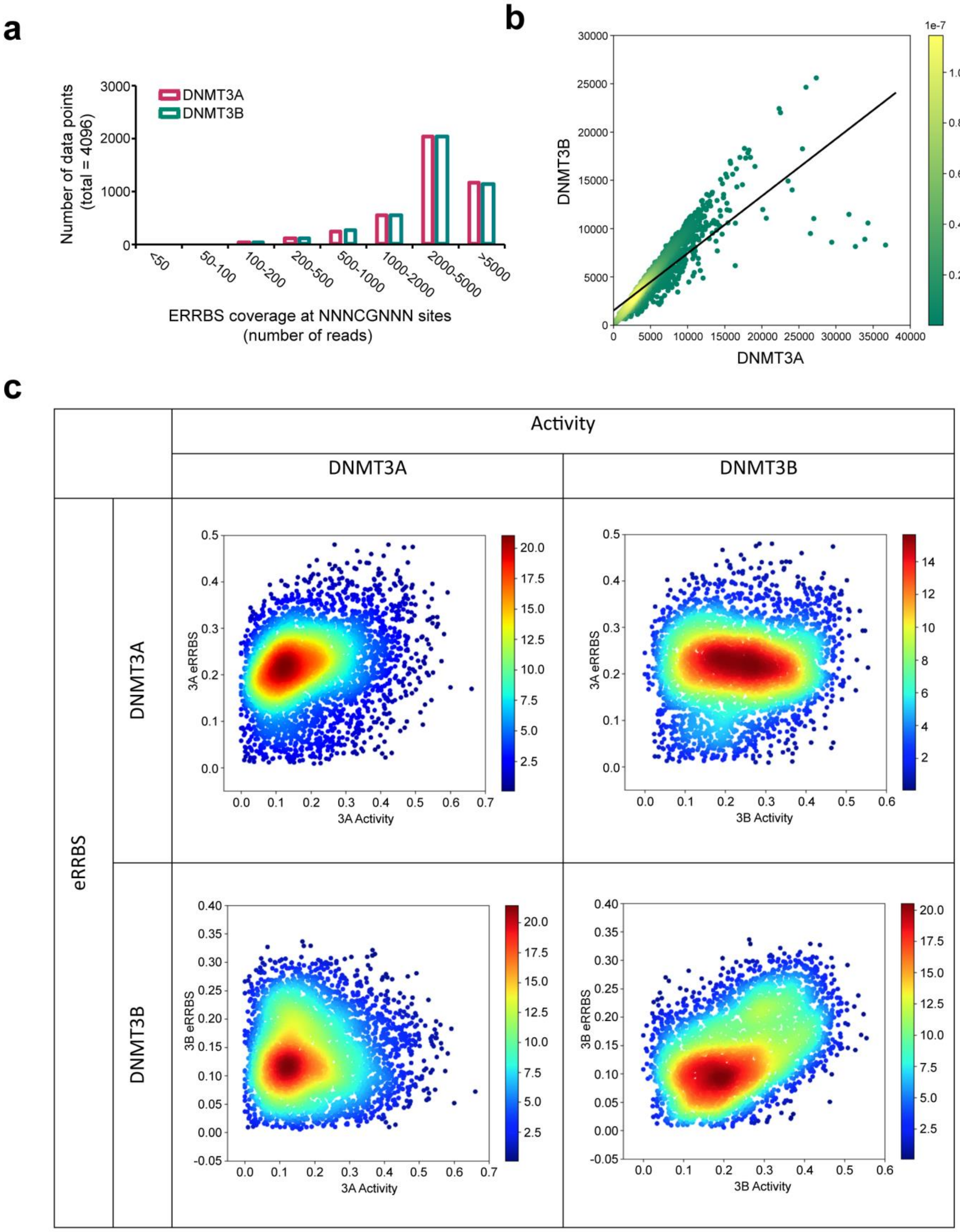

Supplementary Figure 5. Correlation of the NGS-based activity and eRRBS-based methylation level ratios of DNMT3B/DNMT3A. (a,b) Bar (b) and Scatter (c) plots showing the coverage of all 4096 NNNCGNNN sites in the eRRBS analysis of DNMT3B

and DNMT3A based on combined data from two consistent replicates. **(c)** Scatter plot showing the correlation between the eRRBS methylation levels at NNNCGNNN sites introduced by DNMT3A and DNMT3B in ESC TKO cells and NGS-based methylation activities of DNMT3A and DNTM3B.

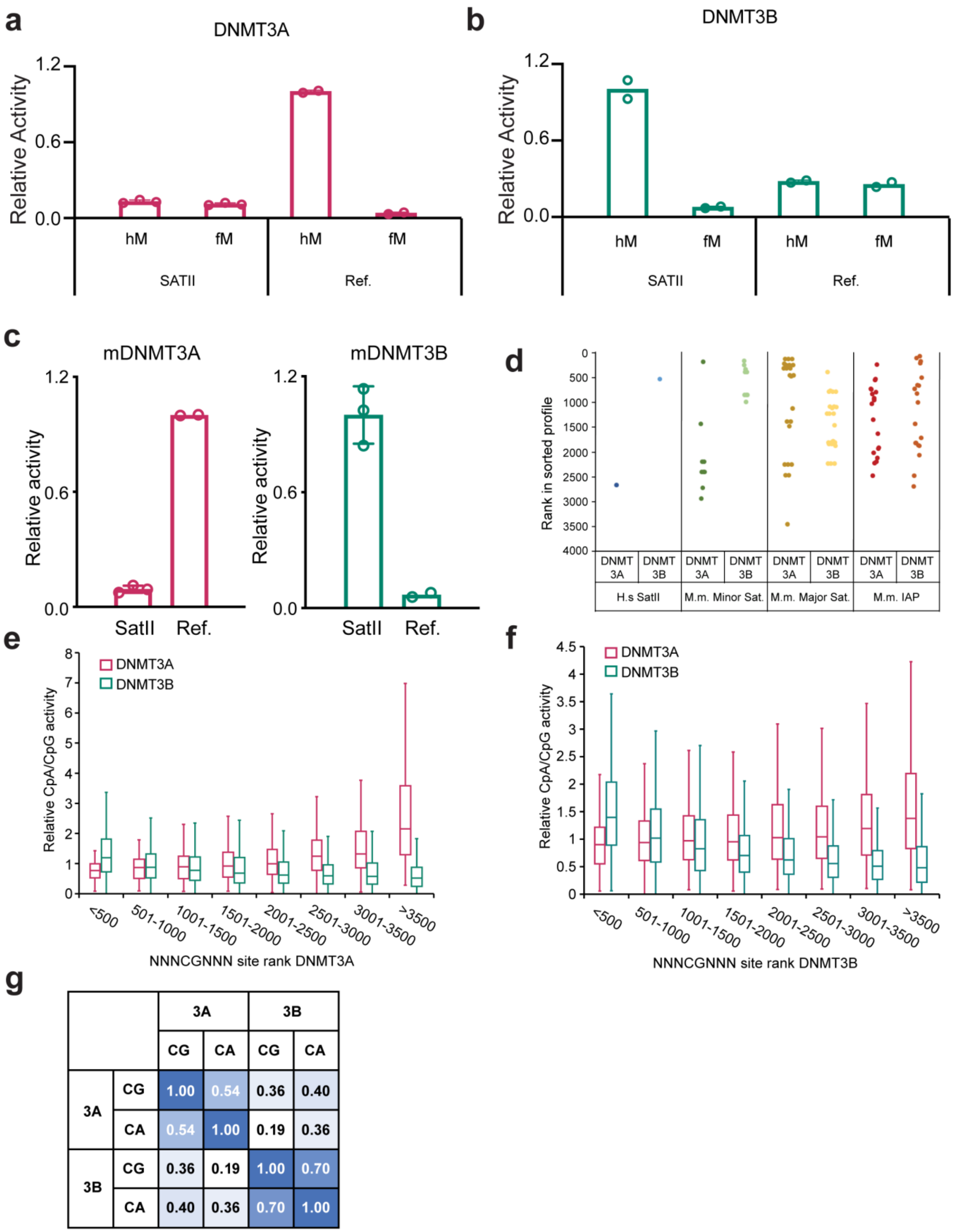

g

		3A		3B	
		CG	CA	CG	CA
3A	CG	1.00	0.54	0.36	0.40
	CA	0.54	1.00	0.19	0.36
3B	CG	0.36	0.19	1.00	0.70
	CA	0.40	0.36	0.70	1.00

Supplementary Figure 6. Additional information regarding the flanking sequence preferences of DNMT3A and DNMT3B. (a,b) Detailed data regarding Fig. 1f.

Enzymatic validation of SatII and reference sequences as a substrate for hDNMT3A (a) or hDNMT3B (b), using the MTase domains of DNMT3A and DNMT3B, respectively, analyzed by radioactive methylation assays. Each substrate was used in hemimethylated form and with fully methylated CpG site and the activity observed with the fully methylated CpG site substrate was subtracted from the methylation detected with the hemimethylated form to specifically determine the methylation of the CpG in the upper DNA strand. n = 2 replicates. **(c)** Experimental validation of the SatII preference of mouse DNMT3B (mDNMT3B) by radioactive methylation assays. The methylation activity of mDNMT3B and mouse DNMT3A (mDNMT3A) was normalized to the more active substrate. Data are mean ± SD of three replicates for SatII substrate and mean of two replicates for reference, respectively. **(d)** Sequence preferences of DNMT3A and DNMT3B for CpG sites in human SatII and mouse repetitive elements. All CpG sites from human SatII repeats (n = 1), mouse minor satellite repeats (Genbank Z22168.1) (n=9), major satellite repeats (Genbank EF028077.1) (n = 23) and IAP elements (Genbank AF303453.1) (n = 19) were retrieved. The figure shows the rank of each site in the mDNMT3A and mDNMT3B preference profiles only considering the more preferred DNA strand. A low rank corresponds to high activity. For average values of DNMT3A/DNMT3B ratios refer to Fig. 2d. (**e,f**) The relative CpA/CpG methylation activity of DNMT3A or DNMT3B as a function of NGS-based substrate preference rank for DNMT3A (e) or DNMT3B (f). The box indicates the 1st and 3rd quartile with mean indicated. Whiskers show the data range. **(g)** Pearson correlation coefficients of CpG and CpA methylation of mDNMT3A and mDNMT3B in NNCGNN flanking sequence context. Note the higher CpG/CpA correlation in mDNMT3B.

a

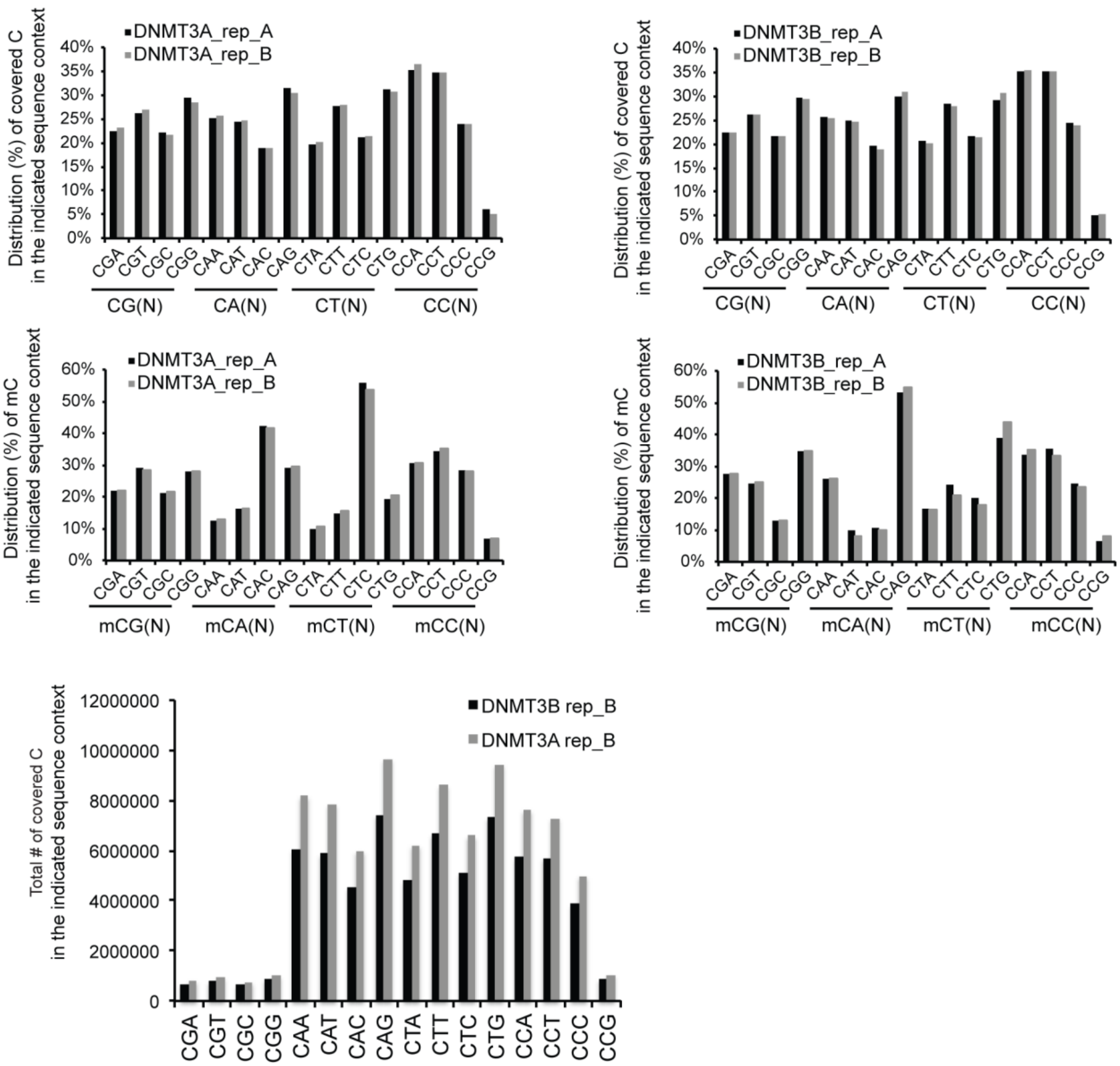

Supplementary Figure 7. Additional controls regarding the context-dependent DNA methylation by DNMT3A and DNMT3B in cells. (a,b) Distribution of the total number of mapped C sites (a) and methylated C (b) with the indicated CNN sequence context in each biological replicate (rep A and B) of the eRRBS methylome profiling of TKO lines rescued with either WT hDNMT3A (left panel) or hDNMT3B (right panel). **(c)** Total counts for the indicated sequence context as covered by the eRRBS-based methylome profiling in cells.

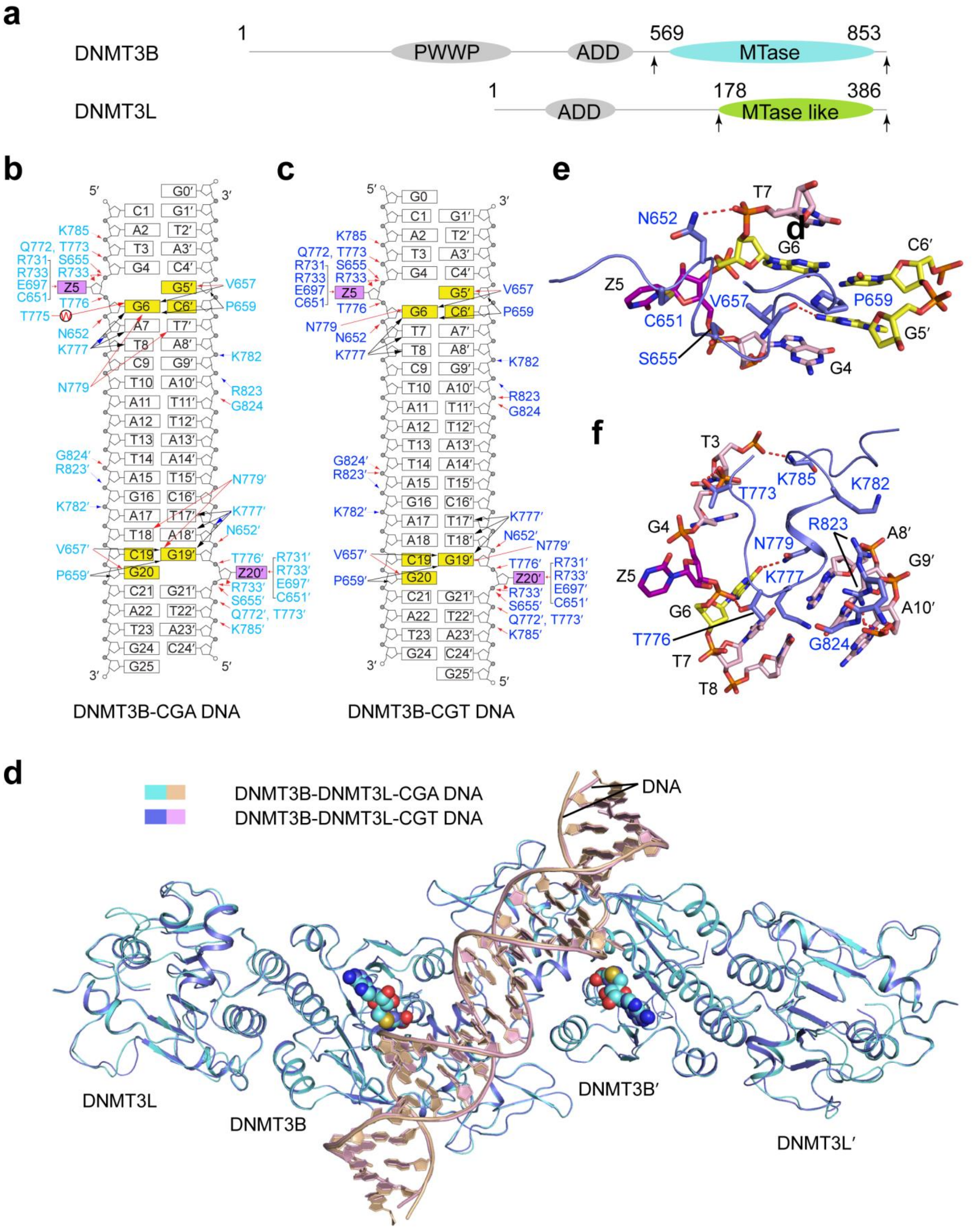

Supplementary Figure 8. Structural details of the DNMT3B-DNMT3L-DNA complexes. (a) Domain architecture of hDNMT3B and hDNMT3L with the C-terminal

domains marked with arrowheads. **(b)** Schematic view of the intermolecular interactions between DNMT3B and CGA DNA. The hydrogen-bonding, electrostatic and van der Waals contacts are represented by red, blue and black arrows, respectively. Water-mediated hydrogen bonds are labeled with letter ‘W’. **(c)** Schematic view of the intermolecular interactions between DNMT3B and CGT DNA. The hydrogen-bonding, electrostatic and van der Waals contacts are represented by red, blue and black arrows, respectively. **(d)** Structural overlay of CGA DNA- (cyan) and CGT DNA-bound (slate) DNMT3B-DNMT3L. The bound DNAs are colored in wheat and light pink, respectively. The SAH molecules are shown in spheres, with carbon atoms colored cyan in the DNMT3B-CGA DNA and slate in the DNMT3B-CGT DNA. **(e-f)** Close-up view of the intermolecular interactions between the catalytic loop (**e**), the TRD loop and a loop at the RD interface (**f**) of DNMT3B and DNA. The hydrogen bonds are shown as dashed lines. The ZpG/CpG sites are colored in purple (Z) or yellow.

		3A		3A3L		3B		3B3L	
		UM	HM	UM	HM	UM	HM	UM	HM
3A	UM	1.00	0.83	0.82	0.78	0.29	0.35	0.28	0.31
	HM	0.83	1.00	0.90	0.90	0.32	0.35	0.30	0.34
3A3L	UM	0.82	0.90	1.00	0.86	0.41	0.40	0.40	0.40
	HM	0.78	0.90	0.86	1.00	0.39	0.46	0.38	0.46
3B	UM	0.29	0.32	0.41	0.39	1.00	0.91	0.92	0.90
	HM	0.35	0.35	0.40	0.46	0.91	1.00	0.88	0.91
3B3L	UM	0.28	0.30	0.40	0.38	0.92	0.88	1.00	0.89
	HM	0.31	0.34	0.40	0.46	0.90	0.91	0.89	1.00

Supplementary Figure 9. Influence of the methylation level of the CpG site and presence of DNMT3L on the flanking sequence preferences of DNMT3A and DNMT3B. Methylation experiments of random flank substrates with unmethylated (UM) or hemimethylated (HM) CpG site were conducted with murine DNMT3A and DNMT3B in the presence of absence of mDNMT3L. Each experiment was performed in two independent repeats, which were highly correlated and merged for this analysis. The figure shows the pairwise Pearson correlation factors of the average methylation levels in each NNCGNN flanking context.

a

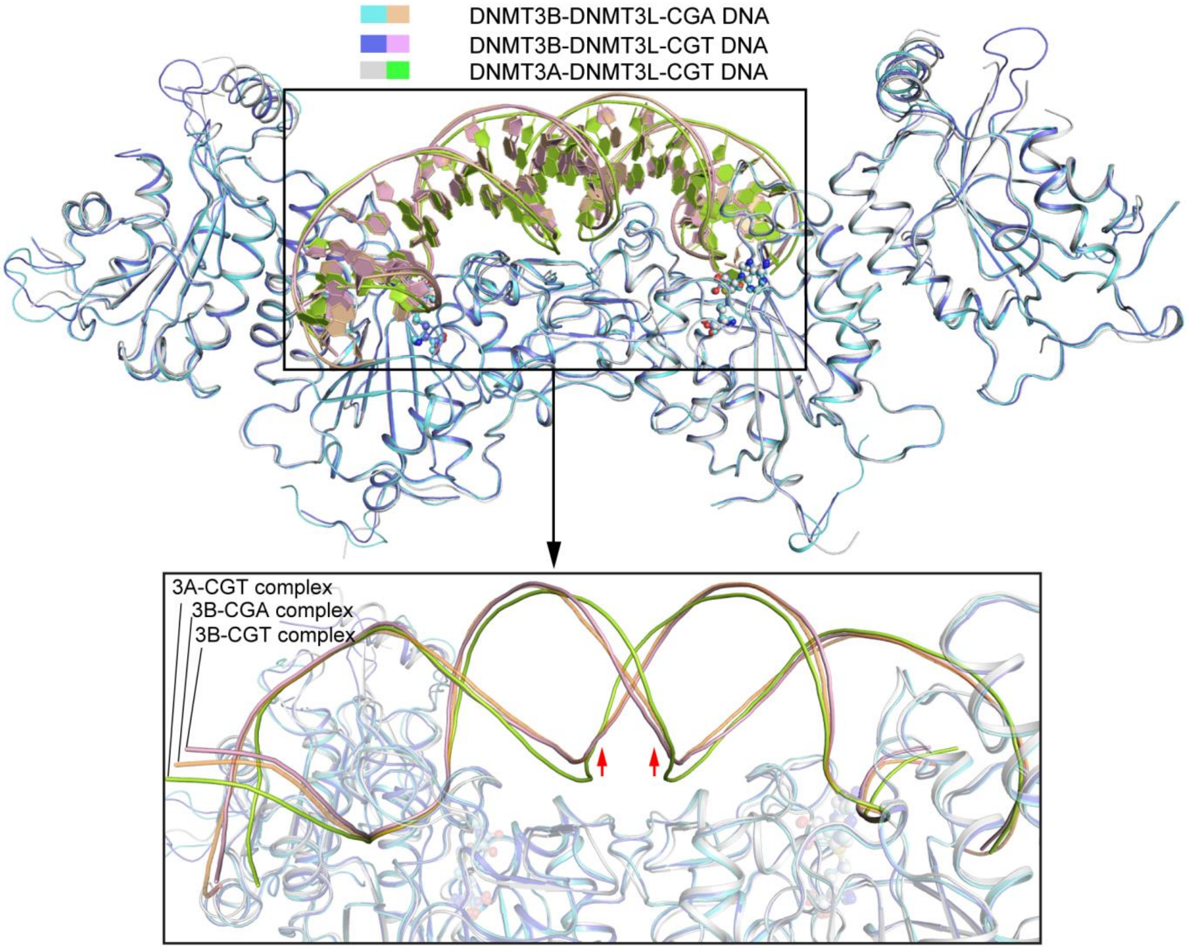

DNMT3B-DNMT3L-CGA DNA
DNMT3B-DNMT3L-CGT DNA
DNMT3A-DNMT3L-CGT DNA
3A-CGT complex
3B-CGA complex
3B-CGT complex

b

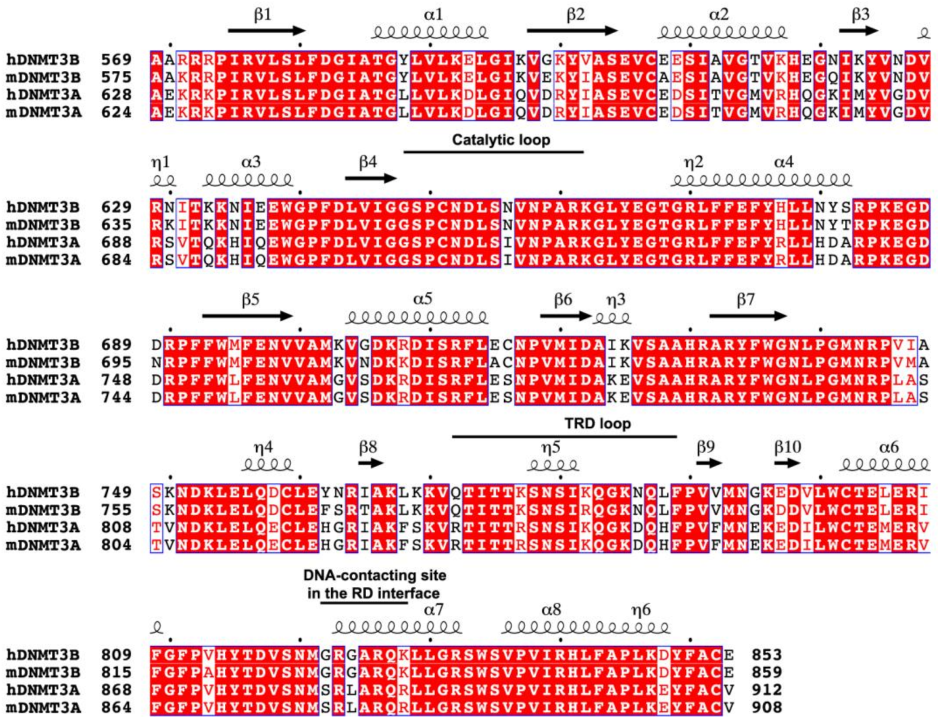

β1 α1 β2 α2 β3
hDNMT3B 569 AARRRPIRVLSLFDGIATGYLVLKELGIKVGKYVASEVCEESIAVGTVKHEGNIKYVNDV
mDNMT3B 575 AAKRRPIRVLSLFDGIATGYLVLKELGIKVEKYIASEVCAESIAVGTVKHEGQIKYVNDV
hDNMT3A 628 AEKRKPIRVLSLFDGIATGLLVLKDLGIQVDRYIASEVCEDSITVGMVRHQGKIMYVGDV
mDNMT3A 624 AEKRKPIRVLSLFDGIATGLLVLKDLGIQVDRYIASEVCEDSITVGMVRHQGKIMYVGDV
Catalytic loop
η1 α3 β4 η2 α4
hDNMT3B 629 RNITKKNIEEWGPFDLVIGGSPCNDLSNVNPARKGLYEGTGRLFFEFYHLLNYSRPKEGD
mDNMT3B 635 RKITKKNIEEWGPFDLVIGGSPCNDLSNVNPARKGLYEGTGRLFFEFYHLLNYTRPKEGD
hDNMT3A 688 RSVTQKHIQEWGPFDLVIGGSPCNDLSIVNPARKGLYEGTGRLFFEFYRLLHDARPKEGD
mDNMT3A 684 RSVTQKHIQEWGPFDLVIGGSPCNDLSIVNPARKGLYEGTGRLFFEFYRLLHDARPKEGD
β5 α5 β6 η3 β7
hDNMT3B 689 DRPFFWMFENVVAMKVGDKRDISRFLECNPVMIDAIKVSAAHRARYFWGNLPGMNRPVIA
mDNMT3B 695 NRPFFWMFENVVAMKVNDKKDISRFLACNPVMIDAIKVSAAHRARYFWGNLPGMNRPVMA
hDNMT3A 748 DRPFFWLFENVVAMGVSDKRDISRFLESNPVMIDAKEVSAAHRARYFWGNLPGMNRPLAS
mDNMT3A 744 DRPFFWLFENVVAMGVSDKRDISRFLESNPVMIDAKEVSAAHRARYFWGNLPGMNRPLAS
TRD loop
η4 β8 η5 β9 β10 α6
hDNMT3B 749 SKNDKLELQDCLEYNRIAKLKKVQTITTKSNSIKQGKNQLFPVVMNGKEDVLWCTELERI
mDNMT3B 755 SKNDKLELQDCLEFSRTAKLKKVQTITTKSNSIRQGKNQLFPVVMNGKDDVLWCTELERI
hDNMT3A 808 TVNDKLELQECLEHGRIAKFSKVRTITTRSNSIKQGKDQHFPVFMNEKEDILWCTEMERV
mDNMT3A 804 TVNDKLELQECLEHGRIAKFSKVRTITTRSNSIKQGKDQHFPVFMNEKEDILWCTEMERV
DNA-contacting site
in the RD interface
α7 α8 η6
hDNMT3B 809 FGFPVHYTDVSNMGRGARQKLLGRSWSVPVIRHLFAPLKDYFACE 853
mDNMT3B 815 FGFPAHYTDVSNMGRGARQKLLGRSWSVPVIRHLFAPLKDYFACE 859
hDNMT3A 868 FGFPVHYTDVSNMSRLARQRLLGRSWSVPVIRHLFAPLKEYFACV 912
mDNMT3A 864 FGFPVHYTDVSNMSRLARQRLLGRSWSVPVIRHLFAPLKEYFACV 908

Supplementary Figure 10. Structural comparison of the DNMT3B-DNMT3L-DNA and DNMT3A-DNMT3L-DNA complexes. (a) Superposition of the DNMT3B-DNMT3L-CGA DNA, DNMT3B-DNMT3L-CGT DNA and DNMT3A-DNMT3L-CGT DNA structures. The lower part shows a close-up view of the DNAs bound to DNMT3B or DNMT3A. **(b)** Sequence alignment of DNMT3 proteins from human (hDNMT3B, hDNMT3A) and mouse (mDNMT3B and mDNMT3A). Secondary structures are shown above the aligned sequences. Fully conserved residues are colored in white and highlighted in red shade. Partially conserved residues are colored in red. The residues involved in DNA binding are marked on top of the aligned sequences.

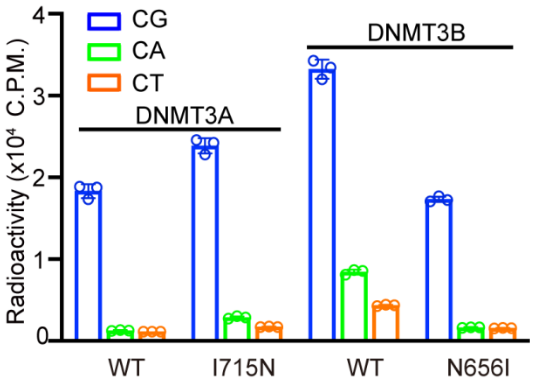

Supplementary Figure 11. Detailed data regarding Fig. 4e. *In vitro* CpG and CpH methylation of hDNMT3A-hDNMT3L and hDNMT3B-hDNMT3L, WT or mutants on the catalytic loop, were measured using $(GAC)_{12}$, $(AAC)_{12}$ and $(TAC)_{12}$ substrates. Data are mean ± SD of three replicates.

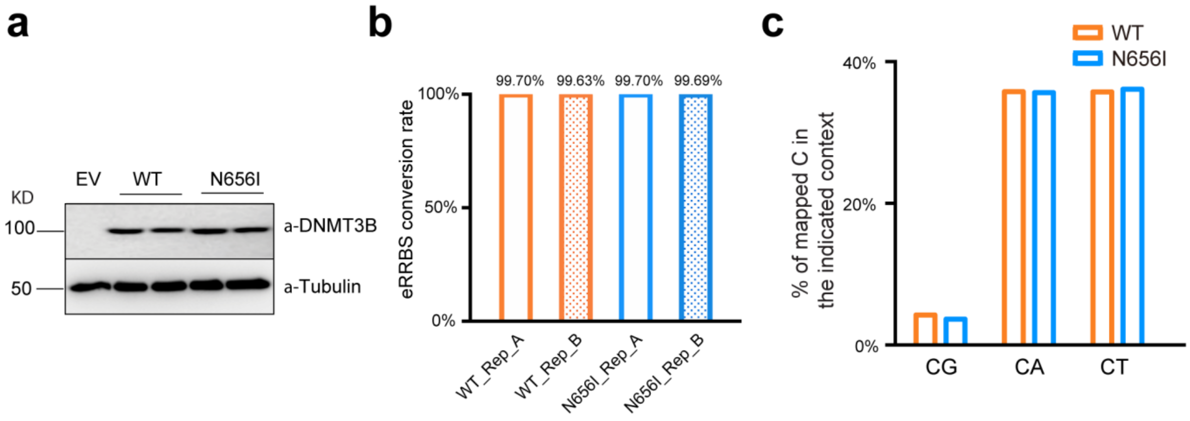

Supplementary Figure 12. Additional data regarding eRRBS methylation profiles of the DNMT3B N656I mutant. (a) Western blot analysis of hDNMT3B, either WT or N656I, post-reconstitution into independently derived TKO lines. **(b)** eRRBS conversion rates of the genomic DNA derived from WT or N656I DNMT3B-transfected TKO cells, as determined by the unmethylated lambda DNA spike-in control. **(c)** eRRBS analysis of WT or N656I hDNMT3B-rescued TKO cells shows similar distribution of the total number of the indicated CpN sites as mapped by sequencing.

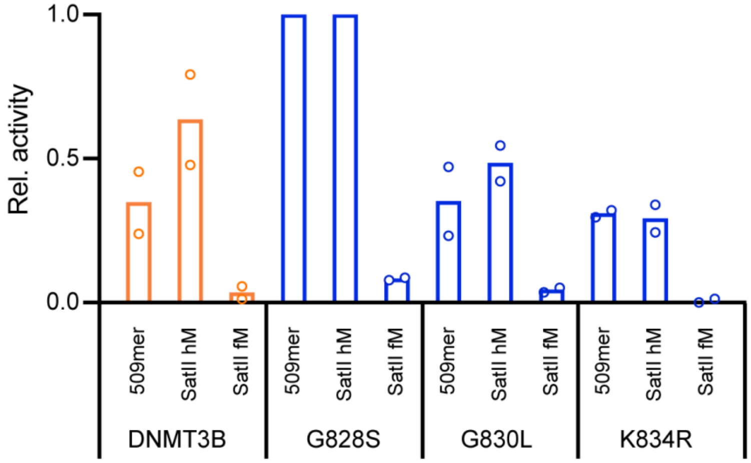

Supplementary Figure 13. Additional data related to Fig. 4i. The mDNMT3B residues G828, G830 and K834 correspond to hDNMT3B G822, G824 and K828. Methylation activities of DNMT3B mutants were determined on the SatII substrate in hemimethylated (SatII hM) and fully methylated form (SATII fM) and a 500-mer DNA with 58 CpG sites used as "neutral" reference substrate (509mer). The relative preference for the SATII site was calculated as ($rate_{SATII\ hM}$-$rate_{SatII\ fM}$)/$rate_{509mer}$ and displayed in relation to WT mDNMT3B (shown in Fig. 4i). The figure shows average values based on two independent measurements.

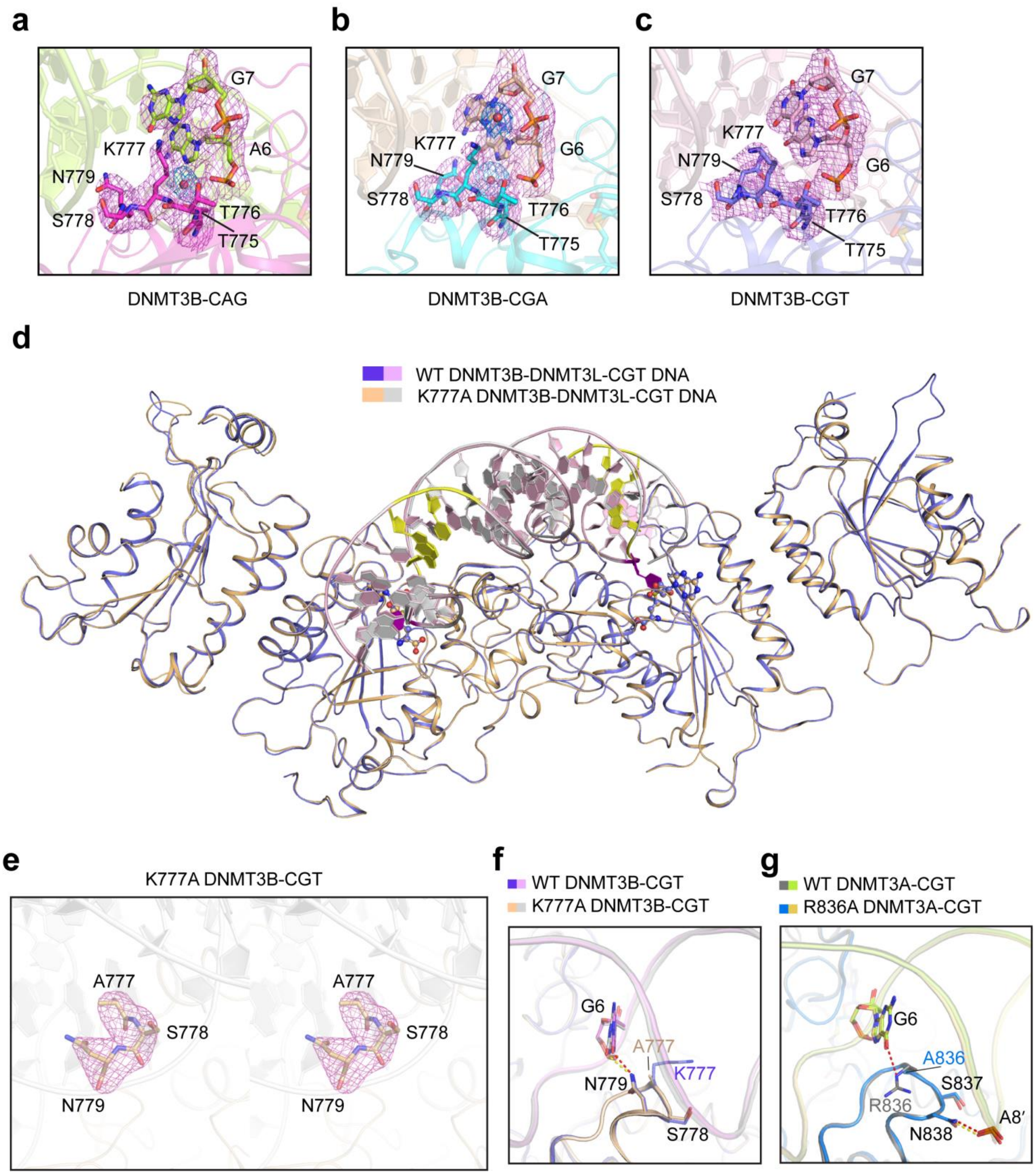

Supplementary Figure 14. Structural analysis of the TRD loop of DNMT3B in DNA-bound form. (a-c) Fo-Fc omit map (violet) for TRD residues T775-N779 of DNMT3B and interacting DNA nucleotides in the DNMT3B-CAG (a), DNMT3B-CGA (b) and DNMT3B-CGT (c) complexes. The Fo-Fc omit map for water molecules (red sphere) are colored blue in (a) and (b). All the omit maps are contoured at 2.0 σ level. **(d)**

Structural superposition of the DNMT3B-DNMT3L-CGT DNA and K777A-mutated DNMT3B-DNMT3L-CGT DNA complexes. The CpG/ZpG sites are colored in purple (Zebularine) or yellow. The SAH molecules are shown in sphere representation. **(e)** Stereo view of Fo-Fc omit map (light magenta), contoured at 2.0 σ level, for TRD residues A777-N779 of K777A-mutated DNMT3B-CGT complex. **(f)** Close-up view of the aligned WT DNMT3B-CGT and K777A-mutated DNMT3B-CGT complexes, with the hydrogen bonds in the WT and K777A complexes shown as red and wheat dashed lines, respectively. **(g)** Close-up view of the aligned WT DNMT3A-CGT (PDB 5YX2) and R836A-mutated DNMT3A-CGT (PDB 6BRR) complexes, with the hydrogen bonds in the WT and R836A complexes shown as red and lemon dashed lines, respectively.

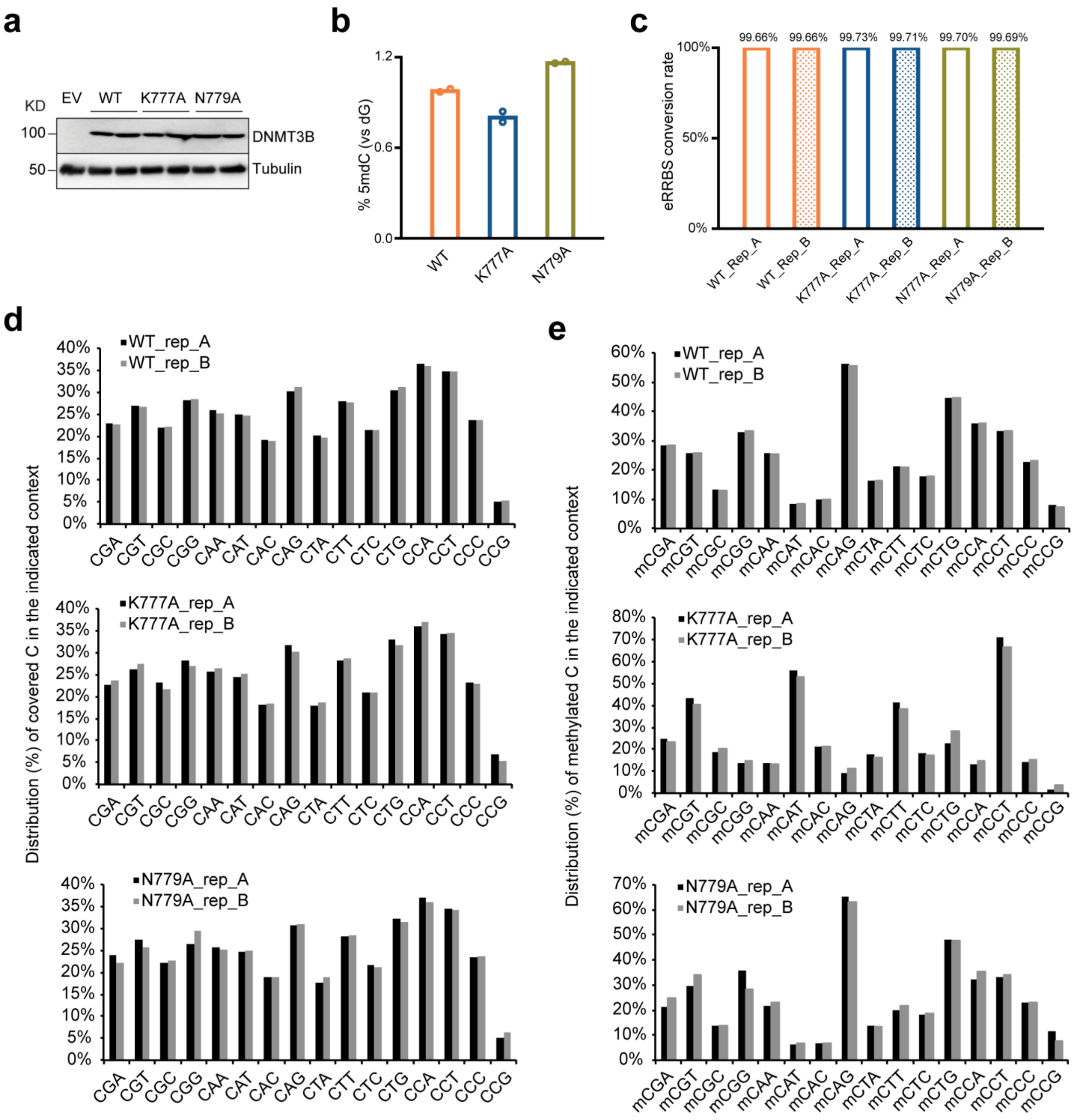

Supplementary Figure 15. Additional data regarding eRRBS methylation profiles of DNMT3B mutants N777A and K779A. (a) Western blot analysis of stably expressed DNMT3B, either WT, K777A or N779A, post-reconstitution into TKO cells. Independently derived lines for each DNMT3B construct were used here and in the following analysis such as eRRBS. **(b)** Liquid chromatography-mass spectrometry (LC-MS) analysis reveals the global 5-mC levels (as indicated by 5-mdC/dG ratio on the y-axis) in the TKO ES cells after stable transduction of EV or the indicated DNMT3B (n = 2 biological replicates). EV, empty vector. **(c)** eRRBS conversion rates of the genomic DNA derived from WT, K777A or N779A DNMT3B-transfected TKO cells, as

determined by the unmethylated lambda DNA spike-in control. **(d,e)** Bar plots show distribution of the total number of mapped C sites (**d**) and methylated C (**e**) with the indicated CNN sequence context in each biological replicate (rep A and B) of TKO lines rescued with hDNMT3B, either WT (top panel) or the K777A- (middle) or N779A-mutated (bottom).

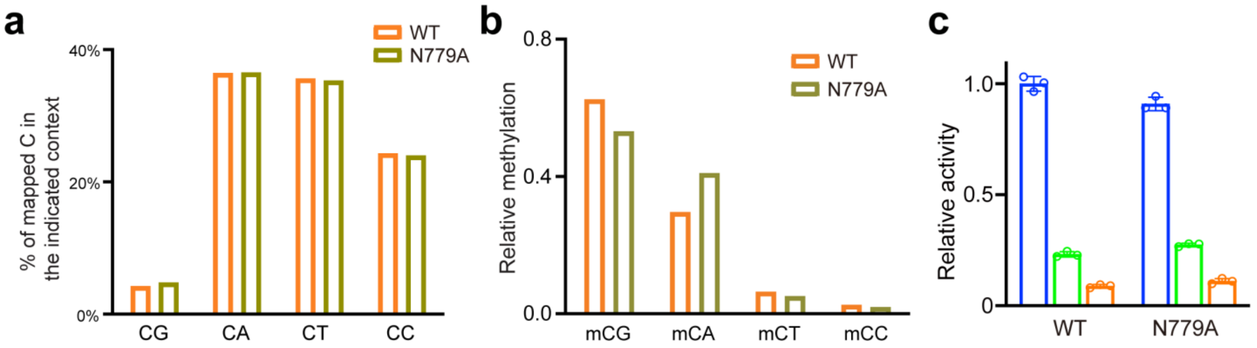

Supplementary Figure 16. eRRBS and enzymatic analyses of the base preference and selectivity of the DNMT3B N779 mutation. (a) eRRBS analysis of WT or N779A hDNMT3B-rescued TKO cells shows similar distribution of the total number of the indicated CpN sites as mapped by sequencing (depth of reads >3 and $p < 0.0001$). **(b)** eRRBS analysis revealing relative methylation of the indicated context in the TKO cells rescued with WT hDNMT3B or N779A (depth of reads >3 and $p < 0.0001$). **(c)** *In vitro* CpG and CpH methylation of DNMT3B-DNMT3L, WT or N779A, using $(GAC)_{12}$, $(AAC)_{12}$ and $(TAC)_{12}$ substrates analyzed by radioactive methylation assays. Data are mean ± SD of three replicates.

Supplemental Table 1: Compilation of Deep enzymology experiments indicating conditions, read counts and average methylation levels.

Enzyme	c (µM)#	Target (in N_{10} context)		Read#	Av. Meth.
**DNMT3A**	0.5	CG (hemimet)		653331	0.209
	1	CG (hemimet)		131110	0.711
**DNMT3B**	2	CG (hemimet)		93991	0.175
	4	CG (hemimet)		951779	0.250
**No enzyme**	-	CG (hemimet)		150155	0.003
**DNMT3A**	20	CH	CA	50745	0.404
			CT	47960	0.126
			CC	45624	0.044
**DNMT3A**	10	CH	CA	69385	0.286
			CT	69094	0.084
			CC	61841	0.027
**DNMT3B**	20	CH	CA	42679	0.092
			CT	43465	0.032
			CC	43445	0.021
**DNMT3B**	10	CH	CA	34769	0.071
			CT	35907	0.029
			CC	34888	0.019
**DNMT3A**	0.5	CN	CG	10305	0.501
			CA	10988	0.022
			CT	11029	0.007
			CC	11343	0.010
**DNMT3B**	10	CN	CG	11923	0.514
			CA	10971	0.067
			CT	10970	0.029
			CC	11338	0.027
**DNMT3B K777A**	10	CN	CG	7487	0.518
			CA	7277	0.141
			CT	7249	0.037
			CC	7472	0.051
**DNMT3A**	0.5, 1	CG (unmet)		94022	0.756
**No enzyme**	-	CG (unmet)		142836	0.002
**DNMT3A DNMT3L**	0.125, 0.25	CG (hemimet)		93675	0.211
**DNMT3A DNMT3L**	0.125, 0.25	CG (unmet)		104185	0.309
**DNMT3B**	1, 2	CG (unmeth)		208584	0.184
**DNMT3B DNMT3L**	0.125, 0.25	CG (hemimet)		111634	0.168
**DNMT3B DNMT3L**	0.125, 0.25	CG (unmet)		70413	0.154

#Some data sets were combined from different experiments to increase the coverage.

Supplementary Table 2: Details of the analysis of the deep enzymology experiments. List of NNNCGNNN sites with coverage >50 and very high (>50%) or very low (<1%) methylation by DNMT3A or DNMT3B.

DNMT3A

Sites with methylation level >50%

TTC CG CCC	0.66
ATC CG CCC	0.60
TTA CG CTC	0.59
ATA CG CCC	0.58
TTC CG CTC	0.57
ATA CG CTT	0.56
ATA CG CCT	0.56
CTA CG CCA	0.56
CTT CG CTC	0.55
ATA CG CTC	0.55
CTA CG CCT	0.55
TTA CG TTA	0.55
TTA CG CTT	0.55
CTC CG CCT	0.55
GTA CG CTC	0.54
CTA CG TCC	0.54
GTC CG TCG	0.54
CTA CG CCC	0.54
ATA CG CCA	0.54
GTA CG CTT	0.53
CCA CG CTC	0.53
TTC CG TCT	0.53
TTG CG CCC	0.53
GTC CG CCC	0.53
TTA CG GGC	0.53
ATC CG CCT	0.53
GTG CG CCT	0.52
CTC CG CCC	0.52
TCA CG CTC	0.52
TTA CG CGC	0.52
CTA CG CCG	0.51
ATC CG TCC	0.51
GCA CG CCC	0.51
TTC CG CCT	0.51
ACA CG CTC	0.51
CTA CG TCG	0.50
ACA CG TCC	0.50
CTC CG TCC	0.50
CTA CG CTA	0.50

Sites with methylation level <1%

GAT CG GAG	0.0098
GAT CG TGG	0.0098
AGG CG TGG	0.0096
GAT CG ATG	0.0096
CGG CG CGG	0.0089
GGA CG TGG	0.0085
GAT CG GGT	0.0083
AGT CG CGG	0.0082
AGT CG TAG	0.0076
CGT CG TGC	0.0074
TAG CG CGG	0.0071
AAT CG TGG	0.0068
AAT CG GAG	0.0042
AAT CG CGG	0.0040
AGG CG GGG	0.0000
CGG CG GAG	0.0000
CGG CG TGG	0.0000
GAG CG TGG	0.0000
GAT CG TGT	0.0000
GGG CG TGG	0.0000
GGT CG GGG	0.0000
GTT CG GGG	0.0000
TAA CG TGG	0.0000
TCT CG TGG	0.0000
TGG CG TGT	0.0000
TGT CG GAG	0.0000
TGT CG GGG	0.0000

DNMT3b

Sites with methylation level >50%

TTC CG ATC	0.55
CTA CG GCT	0.55
TTA CG GCG	0.54
CTA CG GCA	0.53
TTC CG GTT	0.53
CTT CG GGC	0.52
CTA CG AGC	0.51
ATA CG ATC	0.51
AGT CG GCA	0.51
ATA CG GCG	0.51
ATA CG GCA	0.51
TTA CG AGC	0.50

Sites with methylation level <1%

GGC CG TGG	0.0092
AGG CG TTG	0.0088
GGC CG TTG	0.0082
TCC CG TGG	0.0068
TGG CG TGT	0.0000
AGC CG TGG	0.0000

Supplementary Table 3. Summary for the eRRBS-based methylome profiling of TKO cells rescued with WT DNMT3A or DNMT3B, either WT or the N656I mutant. Two independently derived lines were used for each rescue group.

Sample ID	***Read 1; Expect CGG/TGG/TAA/TAG***			***Read 2; Expect CAA/CAG/TAA/TAG***		
	consistent	***total***	***%***	***consistent***	***total***	***%***
DNMT3A_rep1	56,473,302	59,784,389	94.5%	54,361,578	59,784,389	90.9%
DNMT3A_rep2	57,317,416	60,491,707	94.8%	55,718,373	60,491,707	92.1%
DNMT3B_rep1	47,761,659	51,378,043	93.0%	45,312,074	51,378,043	88.2%
DNMT3B_rep2	32,763,933	35,283,821	92.9%	30,183,214	35,283,821	85.5%
DNMT3B_N656I_rep1	77,296,475	83,850,179	92.2%	72,543,591	83,850,179	86.5%
DNMT3B_N656I_rep2	53,574,861	57,963,819	92.4%	49,819,268	57,963,819	85.9%
		min	*92.18%*		*min*	*85.54%*
		max	*94.75%*		*max*	*92.11%*
		mean	*93.27%*		*mean*	*88.21%*
		median	*92.91%*		*median*	*87.35%*

Supplementary Table 4. X-ray data collection and refinement statistics.

	DNMT3B-DNMT3L-CGA DNA (PDB: 6U8P)	DNMT3B-DNMT3L-CGT DNA (PDB: 6U8V)	DNMT3B-DNMT3L-CAG DNA (PDB: 6U8X)	DNMT3B^{K777A}-DNMT3L-CGT DNA (PDB: 6U8W)
**Data collection**				
**Space group**	P 31	P 31	P 31	P 31
**Cell dimensions**				
***a*, *b*, *c* (Å)**	193.3, 193.3, 49.7	194.2, 194.2, 49.7	193.8, 193.8, 49.9	193.5 193.5 49.9
**α, β, γ (°)**	90.00, 90.00, 120.00	90.00, 90.00, 120.00	90.00, 90.00, 120.00	90.00, 90.00 120.00
**Resolution (Å)**	48.33-3.04 (3.15-3.04)	48.55-3.00(3.10-3.00)	42.88-2.95 (3.06-2.95)	48.38-2.95 (3.05-2.95)
**R_{merge}**	0.130 (0.833)	0.107 (0.682)	0.110 (0.994)	0.097 (0.733)
**$I/\sigma(I)$**	8.5 (1.3)	11.8 (2.3)	8.8 (1.2)	7.91 (1.34)
**$CC_{1/2}$**	0.996 (0.843)	0.997 (0.924)	0.998 (0.763)	0.997 (0.782)
**Completeness (%)**	99.8 (99.4)	99.3 (94.2)	99.64 (99.20)	99.7 (99.3)
**Redundancy**	4.3 (4.0)	5.0 (4.9)	5.2 (4.7)	3.4 (3.3)
**Refinement**				
**No. reflections**	39736	41814	43905	43844
**R_{work} / R_{free}**	0.204/0.237	0.204/0.240	0.227/0.252	0.216/0.236
**No. atoms**				
**Protein and DNA**	8398	8527	8521	8380
**Ligand**	52	52	52	52
**Water**	25	11	19	11
***B* factors (Å^2)**				
**DNMT3B**	69.8	68.5	66.2	70.8
**DNMT3L**	120.7	121.7	123.6	127.2
**DNA**	142.7	124.3	124.9	155.2
**Ligand**	54.4	47.2	50.5	78.4
**Water**	61.6	52.1	60.6	65.6
**r.m.s deviations**				
**Bond lengths (Å)**	0.006	0.007	0.003	0.004
**Bond angles (°)**	1.11	1.16	0.62	0.68

[a]Values in parentheses are for highest-resolution shell. Each structure was determined using the dataset collected from a single crystal.

Supplementary Table 5. Summary for the eRRBS-based methylome profiling of TKO cells rescued with DNMT3B, either WT, K777A- or N779A-mutated. Two independently derived lines were used for each rescue group. WT DNMT3B-expressing lines used here differ from the two used in Supplementary Table 3.

Sample id	***Read 1; Expect CGG/TGG/TAA/TAG***			***Read 2; Expect CAA/CAG/TAA/TAG***		
	consistent	***total***	***%***	***consistent***	***total***	***%***
DNMT3B_WT_rep_A	36,433,896	39,005,803	93.4%	35,256,776	39,005,803	90.4%
DNMT3B_WT_rep_B	37,491,113	40,223,639	93.2%	36,457,152	40,223,639	90.6%
DNMT3B_K777A_rep_A	68,556,666	75,600,986	90.7%	66,547,716	75,600,986	88.0%
DNMT3B_K777B_rep_B	76,653,297	86,775,951	88.3%	74,898,050	86,775,951	86.3%
DNMT3B_N779A_rep_A	52,695,272	58,470,964	90.1%	51,090,356	58,470,964	87.4%
DNMT3B_N779A_rep_B	51,519,467	55,954,185	92.1%	50,711,702	55,954,185	90.6%
		min	*88.34%*		*min*	*86.31%*
		max	*93.41%*		*max*	*90.64%*
		mean	*91.30%*		*mean*	*88.90%*
		median	*91.38%*		*median*	*89.21%*
