# Supplementary material for: Comprehensive structure-function characterization of DNMT3B and DNMT3A reveals distinctive de novo DNA methylation mechanisms
Source: Nat Commun. 2020 Jul 3;11:3355. doi: 10.1038/s41467-020-17109-4 (PMC7335073; doi:10.1038/s41467-020-17109-4)
Supplement: Supplementary file 2 — Reporting Summary [file 41467_2020_17109_MOESM2_ESM.pdf]

nature research

Corresponding author(s): Jikui Song, Albert Jeltsch, Gang Greg Wang

Last updated by author(s): May 31, 2020

Reporting Summary

Nature Research wishes to improve the reproducibility of the work that we publish. This form provides structure for consistency and transparency in reporting. For further information on Nature Research policies, see Authors & Referees and the Editorial Policy Checklist .

Statistics

For all statistical analyses, confirm that the following items are present in the figure legend, table legend, main text, or Methods section.

n/a	Confirmed	
☐	☒	The exact sample size (n) for each experimental group/condition, given as a discrete number and unit of measurement
☐	☒	A statement on whether measurements were taken from distinct samples or whether the same sample was measured repeatedly
☐	☒	The statistical test(s) used AND whether they are one- or two-sided<br>*Only common tests should be described solely by name; describe more complex techniques in the Methods section.*
☒	☐	A description of all covariates tested
☒	☐	A description of any assumptions or corrections, such as tests of normality and adjustment for multiple comparisons
☐	☒	A full description of the statistical parameters including central tendency (e.g. means) or other basic estimates (e.g. regression coefficient) AND variation (e.g. standard deviation) or associated estimates of uncertainty (e.g. confidence intervals)
☐	☒	For null hypothesis testing, the test statistic (e.g. F, t, r) with confidence intervals, effect sizes, degrees of freedom and P value noted<br>*Give P values as exact values whenever suitable.*
☒	☐	For Bayesian analysis, information on the choice of priors and Markov chain Monte Carlo settings
☒	☐	For hierarchical and complex designs, identification of the appropriate level for tests and full reporting of outcomes
☐	☒	Estimates of effect sizes (e.g. Cohen's d, Pearson's r), indicating how they were calculated

Our web collection on statistics for biologists contains articles on many of the points above.

Software and code

Policy information about availability of computer code

Data collection	X-ray diffraction data were collected from synchrotron beamline BL5.0.1 and BL5.0.2 at Advanced Light Source (ALS), Lawrence Berkeley National Laboratory Laboratory using the standard software of the beamline.
Data analysis	For structural study, the HKL2000, XDS, PHENIX 1.15.2-3472-000, WinCoot 0.8.9 and Pymol v0.99 softwares were used for data processing and analysis. For enzymatic analysis, Microsoft Excel 2010 was used. For Bioinformatic analysis of NGS data, software tools on the Usegalaxy.eu server and home written programs were used. For eRRBS analysis, FastQC v0.11.2, Cutadapt v1.2.1, Bismark v0.18.1, abd Bowtie v1.2 software packages were used. Custom scripts used for NGS and eRRBS analyses are available upon request.

For manuscripts utilizing custom algorithms or software that are central to the research but not yet described in published literature, software must be made available to editors/reviewers. We strongly encourage code deposition in a community repository (e.g. GitHub). See the Nature Research guidelines for submitting code & software for further information.

Data

Policy information about availability of data

All manuscripts must include a data availability statement. This statement should provide the following information, where applicable:

- Accession codes, unique identifiers, or web links for publicly available datasets
- A list of figures that have associated raw data
- A description of any restrictions on data availability

Coordinates and structure factors for the DNMT3B complexes have been deposited in the Protein Data Bank under accession codes 6U8P, 6U8V, 6U8X and 6U8W. The deep enzymology data have been deposited in the data repository of the University of Stuttgart DARUS (https://darus.uni-stuttgart.de/) under https://doi.org/10.18419/darus-627. The eRRBS data have been deposited in Gene Expression Omnibus (GEO) with the accession number GSE145899. Additional database entries used in this study: Genebank Z22168.1 (M.musculus minor satellite DNA; https://www.ncbi.nlm.nih.gov/nuccore/Z22168.1), EF028077.1 (Mus musculus isolate 022 major satellite repeat sequence; https://www.ncbi.nlm.nih.gov/nuccore/EF028077), AF303453.1 (Mus musculus endogenous virus intracisternal A-particle; https://www.ncbi.nlm.nih.gov/nuccore/AF303453.1), NC_001416.1 (Enterobacteria phage lambda, complete genome; https://www.ncbi.nlm.nih.gov/nuccore/NC_001416.1), NM_006892 (human DNMT3B: https://www.ncbi.nlm.nih.gov/nuccore/NM_006892.3), NM_022552 (human DNMT3A: https://

www.ncbi.nlm.nih.gov/nuccore/NM_022552), PDB 5YX2 (Crystal structure of DNMT3A-DNMT3L in complex with CGT DNA; https://www.rcsb.org/structure/5yx2), and GRCm38 (Mus musculus genome sequence mm10; https://www.ncbi.nlm.nih.gov/assembly/GCF_000001635.20). The sequencing data underlying Fig. 1a-d, Fig. 2a and b, and Supplementary Figs 2, 5, 6e-g and 9 are available at the data repository of the University of Stuttgart DARUS (https://darus.uni-stuttgart.de/) under https://doi.org/10.18419/darus-627. The source data underlying Figs 1a,e,f, 2a,b, 3f, 4a,d,e,i, 7a,b, and Supplementary Figs 3a,b, 4b, 6a-c, 12a, 13, 15a,b, 16c are provided as a Source Data file.

Field-specific reporting

Please select the one below that is the best fit for your research. If you are not sure, read the appropriate sections before making your selection.

☒ Life sciences ☐ Behavioural & social sciences ☐ Ecological, evolutionary & environmental sciences

For a reference copy of the document with all sections, see nature.com/documents/nr-reporting-summary-flat.pdf

Life sciences study design

All studies must disclose on these points even when the disclosure is negative.

Sample size	Biochemical and enzymatic assays were completed using wild type or mutants of DNMT3A, DNMT3B, DNMT3A-DNMT3L and DNMT3B-DNMT3L. eRRBS assays were completed using wild type or mutant DNMT3A and DNMT3B plasmids. The sample size is sufficient to delineate the mutational effects of DNMT3A and DNMT3B.
Data exclusions	No data exclusion.
Replication	For deep enzymology assays, two independent measurements were performed and stated in figure legends. For all other in vitro DNA methylation assays, three independent measurements were performed for each sample and stated in figure legends.<br>For cellular and genomics assays, at least 3 biological replicates were used and stated in figure legends. Data are presented as the mean ± SD of at least two independent experiments. Statistical analysis was performed with Student's t test for comparing two sets of data with assumed normal distribution. A p value of less than 0.05 was considered to be significant. All attempts at replication were successful.
Randomization	The assays performed in this study require a rational approach for activity comparison. Therefore, randomization is not applicable to our experimental set up.
Blinding	Blinding is not applicable to any biochemical or cellular assay performed in this study.

Reporting for specific materials, systems and methods

We require information from authors about some types of materials, experimental systems and methods used in many studies. Here, indicate whether each material, system or method listed is relevant to your study. If you are not sure if a list item applies to your research, read the appropriate section before selecting a response.

Materials & experimental systems

n/a	Involved in the study
☐	☒ Antibodies
☐	☒ Eukaryotic cell lines
☒	☐ Palaeontology
☒	☐ Animals and other organisms
☒	☐ Human research participants
☒	☐ Clinical data

Methods

n/a	Involved in the study
☒	☐ ChIP-seq
☒	☐ Flow cytometry
☒	☐ MRI-based neuroimaging

Antibodies

Antibodies used	Antibodies used for immunoblotting include DNMT3A (Abcam ab2850), DNMT3B (Santa Cruz bio., G-9: sc-376043) and α-Tubulin (Sigma, catalog. # T5168).
Validation	Dnmt3a Antibody detects DNA methyltransferase 3a (Dnmt3a) from human and mouse tissues and cells as well as recombinant human Dnmt3a. This antibody does not detect full length recombinant human Dnmt3b or Dnmt1. (See https://www.abcam.com/dnmt3a-antibody-chip-grade-ab2850.html)<br>Dnmt3b Antibody (G-9) is a high quality monoclonal Dnmt3b antibody (also designated DNMT3b antibody, or DNA methyltransferase 3B antibody) suitable for the detection of the Dnmt3b protein of mouse, rat and human origin. (See https://www.scbt.com/p/dnmt3b-antibody-g-9)<br>α-Tubulin recognizes an epitope located at the C-terminal end of the α-tubulin isoform in a variety of organisms. It has been used in immunofluorescence Analysis, estern blotting/ Immunoblotting and for immunolabelling cells in electron microscopy. (See https://www.sigmaaldrich.com/catalog/product/sigma/t5168?lang=en&region=US)

Eukaryotic cell lines

Policy information about cell lines

Cell line source(s)	The mouse embryonic stem cell line lacking DNMTs (Dnmt1-/- Dnmt3a-/- Dnmt3b-/- or TKO-ESCs; a gift from Dr. M. Okano) were cultivated.
Authentication	Authentication of cell line identity, including that of parental and derived lines, was ensured by Tissue Culture Facility affiliated to the Lineberger Comprehensive Cancer Center of UNC at Chapel Hill using the genetic signature profiling and fingerprinting analysis.
Mycoplasma contamination	Every 1-2 month, a routine examination of cell lines in culture for any possible mycoplasma contamination was carried out using MycoAlert Mycoplasma Detection Kit (Lonza). No mycoplasma contamination was identified.
Commonly misidentified lines (See ICLAC register)	No commonly misidentified cell lines were used in the study.
