# Supplementary material for: Comprehensive structure-function characterization of DNMT3B and DNMT3A reveals distinctive de novo DNA methylation mechanisms
Source: Nat Commun. 2020 Jul 3;11:3355. doi: 10.1038/s41467-020-17109-4 (PMC7335073; doi:10.1038/s41467-020-17109-4)
Supplement: Supplementary file 3 — Description of Additional Supplementary Files [file 41467_2020_17109_MOESM3_ESM.pdf]

Description of Additional Supplementary Files

File name: Supplementary Data 1
Description: Deep enzymology and eRRBS analyses of mDNMT3A and mDNMT3B in NNNCGNNN sequences.
